# Two cases of *Leclercia adecarboxylata* septic arthritis in immunocompetent paediatric patients

**DOI:** 10.1099/acmi.0.000325

**Published:** 2022-02-28

**Authors:** Ramesh Arasu, Yean Hsiang Ewe, Abavasankar Sundaram, David Anthony Foley, Anita Jane Campbell, Timothy Robin William Fletcher, Pamela Palasanthiran, Arnold Suzuki

**Affiliations:** ^1^​ School of Medicine, University of Western Australia, Nedlands, Western Australia, Australia; ^2^​ Infectious Diseases, Perth Children’s Hospital, 15 Hospital Avenue, Nedlands 6009, Western Australia, Australia; ^3^​ Wesfarmers Centre of Vaccines and Infectious Diseases, Telethon Kids Institute, Perth, Western Australia, Australia; ^4^​ Department of Orthopaedic Surgery, Bunbury Regional Hospital, Bussell Highway, Bunbury 6230, Australia; ^5^​ Department of Immunology and Infectious Diseases, Sydney Children’s Hospital Network, High Street, Randwick 2031, New South Wales, Australia; ^6^​ School of Women’s and Child Health, University of New South Wales, Kensington, Sydney 2052, New South Wales, Australia; ^7^​ Department of Orthopaedic Surgery, Sydney Children’s Hospital, High Street, Randwick 2031, New South Wales, Australia

**Keywords:** immunocompetent, infection, *Leclercia adecarboxylata*, septic arthritis

## Abstract

*
Leclercia adecarboxylata* is a rare cause of septic arthritis in children, and has intrinsic resistance to common antibiotics. We describe two cases of *L. adecarboxylata* septic arthritis in children that required re-presentation to hospital with prolonged treatment, and highlight the importance of considering *L. adecarboxylata* as a potential cause of infection among children with penetrating injuries and associated environmental exposure.

## Introduction

*Leclercia adecarboxylata* is a Gram-negative bacillus found ubiquitously in the environment, including saltwater, soil and animals, and as a commensal in the human gastrointestinal tract [1]. Although it is considered to be rarely pathogenic, there have been several recent case reports of *L. adecarboxylata* infection in children who have no noted degree of immunocompromise [2–4]. This rise in cases is also attributed to previous laboratory misidentification of *L. adecarboxylata* as *Escherichia coli *, due to a similar biochemical reaction phenotype, rather than a true rise in the frequency of infection. *L. adecarboxylata* was initially assigned to the genus *Escherichia *. It was subsequently reclassified with the emergence of higher-resolution identification techniques in the late 1980s. Notably, modern methods are able to test for further biochemical reactions specific to *L. adecarboxylata *, including production of β-glucosidase, yellow pigment, malonate assimilation, adonitol fermentation, d-arabitol and d-cellobiose, and a negative response to lysine decarboxylase and ornithine decarboxylase testing [5]. Additionally, *L. adecarboxylata* has been found to have inherent resistance to fosfomycin, unlike other *Enterobacteriaceae* [6]. However, these methods were not routinely in use in clinical laboratories in the past, resulting in frequent misnaming of *L. adecarboxylata *. The more recent widespread adoption of matrix-assisted laser desorption/ionization-time of flight MS has simplified access to reliable identification, contributing to the observed rise in rates of infection.

The present report outlines the cases of two healthy children with no significant medical history, who contracted septic arthritis of the knee caused by *L. adecarboxylata *. Initial irrigation, wound closure and antibiotic regimens were ineffective, in part due to resistance to first-generation cephalosporins. These cases demonstrate examples of pathogenic *L. adecarboxylata* in immunocompetent children and the intrinsic resistance of *L. adecarboxylata* against common first-line antibiotics.

## Case 1

A 7-year-old child presented to the emergency department of a hospital in Perth, Western Australia with swelling and limited range of motion of their right knee following an injury sustained 1 day prior. They had fallen with a flexed knee, onto the edge of a wooden garden wall and sustained a 2 cm laceration, 3 cm proximal to the superior pole of their right patella along the midline. On day 1 of the initial presentation, the wound was irrigated superficially, closed with an interrupted monofilament suture, and the child was discharged on oral cephalexin at a dose of 25 mg/kg, four times daily. However, the child re-presented to the hospital the next day due to limited mobility.

**Fig. 1.**
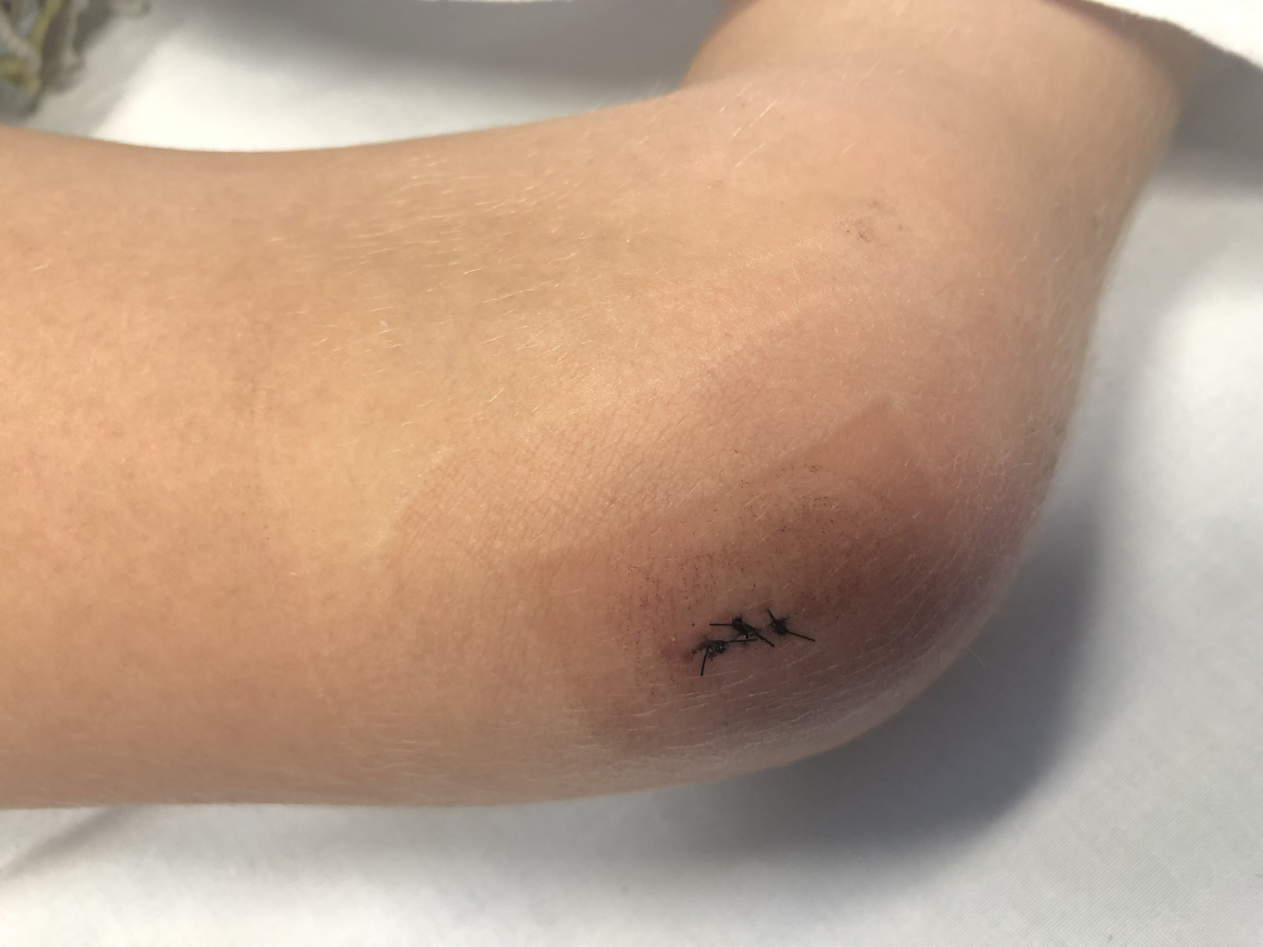
Gross view of the patient’s right knee on return to hospital on day 2 (case 1).

On examination, the child was afebrile with vital signs within normal limits. Their knee was held flexed at 80 °, and they were unable to weight bear or straight leg raise. Passive knee flexion was limited from 30 to 80 °. Their knee was mildly swollen, but the wound site appeared clean (Fig. 1).

**Fig. 2.**
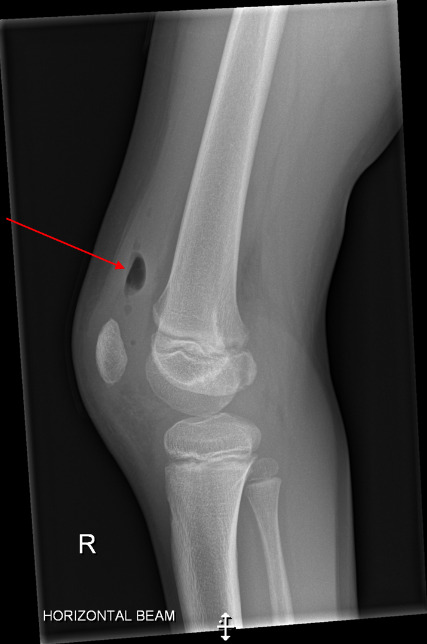
Lateral X-ray of the patient’s right knee showing gas in the suprapatellar pouch on day 2 (case 1).

Laboratory results performed on day 2 showed a slightly raised C reactive protein (CRP) level of 10 mg l^−1^, and a full blood count measuring all cell lines within normal ranges. A lateral radiograph illustrated gas within the joint localized in the suprapatellar region, raising concern of inoculation of the joint due to a penetrating injury. There was no evidence of a fracture, foreign body or osteomyelitis on anterior or lateral X-rays (Fig. 2).

On day 2, the patient was commenced on intravenous (IV) cefazolin, at a dose of 25 mg/kg, 6-hourly. They underwent aggressive surgical debridement on day 3, which revealed contamination of the wound with dark particulate matter suspected to be soil. Following wound closure, antibiotics were changed to IV piperacillin/tazobactam, at a dose of 100 mg/kg every 8 hours. The patient developed diarrhoea as a likely side-effect of the antibiotics. Stool tests for *Clostridium difficile* were negative.

A culture of synovial fluid taken intra-operatively grew *L. adecarboxylata *, which was resistant to cefazolin, but sensitive to other antibiotics including amoxicillin, ceftriaxone, cotrimoxazole and amoxicillin/clavulanate. The antibiotic regimen was changed to IV ceftriaxone at a dose of 50 mg/kg 12-hourly for 7 days, followed by oral amoxicillin at a dose of 25 mg/kg every 8 hours for 3 weeks. A plain radiograph on conclusion of antibiotic therapy showed resolution of previous signs of inoculation, and no signs of further complications.

## Case 2

A 3-year-old patient presented to a hospital emergency department in Sydney, New South Wales with left knee pain after falling onto a tree stump earlier that day. A minor graze was present over the left patella with tenderness and a mild effusion noted on palpation. Their leg was placed in a back-slab and the patient admitted for observation. On day 1, they developed a fever of 38.7 °C and blood results showed raised neutrophils (9.4×10^9^ cells l^−1^) and CRP (69.5 mg l^−1^). On day 3, the patient’s CRP increased to 180 mg l^−1^ and an ultrasound of the left knee joint revealed a 20 ml effusion. The patient was transferred to a tertiary hospital and taken to theatre on day 4 for an arthroscopic washout and drain insertion. Post-operatively, they were commenced on IV cefazolin, at a dose of 25 mg/kg every 6 hours. A culture of synovial fluid taken intra-operatively grew *L. adecarboxylata* that was sensitive to ampicillin and cefotaxime. Antibiotics were changed to IV ceftriaxone at a dose of 100 mg/kg on day 13 for a daily antibiotic regimen for ease of management. However, the patient remained persistently febrile, with left knee swelling and pain. A second arthroscopic washout with partial synovectomy was performed on day 13. A foreign body was located within the synovium and removed from the knee joint. The patient improved both clinically and biochemically. 16S rRNA identification from the foreign body in the second washout included *Citrobacter koseri *, *Escherichia vulneris* and *Bacillus cereus *. The patient was treated with oral ciprofloxacin at a twice-daily dose of 15 mg/kg for 7 weeks with resolution of symptoms.

However, the patient again developed pain and swelling of the left knee 1 month following cessation of antibiotics (day 77). An X-ray of the left knee revealed a large joint effusion. MRI (magnetic resonance imaging) showed a thickened synovium consistent with aseptic arthritis, but no evidence of osteomyelitis. A third washout was performed along with a synovectomy and 15 ml bloodstained straw-coloured fluid was drained. Visible foreign bodies were not seen on this washout. Bacterial, fungal and mycobacterial cultures were negative. IV ceftriaxone was commenced at a once-daily dose of 100 mg/kg for 11 days before switching to oral cephalexin at a dose of 25 mg/kg four times daily to complete a 6 week course of antibiotic therapy. In total, this patient had 12 weeks of antibiotic treatment, and had complete resolution of clinical signs and symptoms at the end of therapy.

## Discussion

The clinical spectrum of disease caused by *L. adecarboxylata* described in the literature is variable, ranging from soft-tissue infections, bacteraemia, urinary tract infections, septic arthritis, osteomyelitis and peritonitis. It is a rare pathogen in immunocompetent patients, where it is frequently associated with polymicrobial infections, as is described in the present cases [7]. Importantly, a number of cases in adult patients have demonstrated fatal outcomes including sepsis and death from multi-organ failure [8].

Whilst there have been over 70 cases described of *L. adecarboxylata* infection in immunocompromised adults, there have only been 3 in immunocompetent children [7]. In 2015, a case report detailed a 2-year-old boy with a superficial wound infection associated with a foreign body that cultured *L. adecarboxylata* [2]. Also in 2015, there was a 9-year-old girl who presented with persistent pain 2 months after surgery for a foot laceration associated with a foreign body, and developed a wound infection culturing *L. adecarboxylata* [3]. She required three different courses of antibiotics and a second surgical debridement to remove a persisting splinter fragment. In 2018, a 9-year-old boy had a positive culture for *L. adecarboxylata* following a laceration to his leg [4]. He underwent an initial washout and debridement, and was discharged on a 7 day course of oral cephalexin. However, he re-presented with necrosis and pyogenic abscess formation, requiring 5 days of washouts and dressings, as well as broad-spectrum antibiotics. All three of these patients eventually recovered following surgery for removal of foreign objects and targeted effective antibiotic therapy. Two of these patients did, however, require multiple courses of antibiotics, and experienced prolonged pain and extended hospital admissions (Table 1).

**Table 1.** Reported *L. adecarboxylata* infections in immunocompetent children

Author/Year	Age (years)	Sex	Mechanism of injury	Site of infection	Treatment used	Final outcome
Hurley *et al.* 2015 [2]	2	Male	Laceration from paper	Thumb wound	Surgical debridement, amoxycillin/clavulanate, ampicillin/sulbactam, amoxycillin	Improvement
Grantham *et al.* 2015 [3]	9	Female	Penetrating injury from environmental debris	Foot wound	Surgical debridement, cephalexin, sulfamethoxazole/trimethoprim, cefepime, levofloxacin	Improvement
Capretta et al. 2018 [4]	9	Male	Penetrating injury from tree trunk	Leg wound	Surgical debridement, cephalexin, piperacillin/tazobactam, ampicillin/sulbactam, amoxycillin/clavulanate	Improvement

The two cases described here represent further examples of *L. adecarboxylata* causing clinically significant disease in immunocompetent children. The importance of eliciting any preceding trauma in the setting of possible osteoarticular infection, particularly associated with a skin breach in contact with the outdoor environment, is demonstrated here. Any wound around joints where the soft-tissue envelope is thin, such as the knee or ankle, should carry a high suspicion of traumatic inoculation. Gas in the joint or soft tissues, or an effusion, is suggestive of an infection. This should raise clinical suspicion of the possibility of foreign-body-associated infection, even in the absence of systemic signs such as fever, and direct further imaging and early operative joint visualization and washout to importantly achieve: (i) source control, and (ii) provide direction for targeted therapy with microbiological, fungal and mycobacterial cultures.

As with our cases, *L. adecarboxylata* is intrinsically resistant to first-generation cephalosporins and semi-synthetic penicillins, which are recommended first-line agents for empirical treatment of paediatric osteoarticular infection in Australia. *L. adecarboxylata* is typically susceptible to most other antibiotics; however, there have been cases of multi-drug-resistant strains [9]. Treating clinicians should consider, in settings with significant preceding environmental trauma, broad-spectrum empirical therapy that still covers for the most frequently identified organisms

### Conclusion

*L. adecarboxylata* infection is rare among patients, and rarer still among immunocompetent children. Its re-classification as distinct from the genus *Escherichia *, and the adoption of modern laboratory techniques, have likely contributed to an increased number of case reports in recent years. Nevertheless, it may represent an important emerging pathogen, in part due to its potential for developing antibiotic resistance [6]. The association with penetrating injuries and the need to seek and remove residual foreign bodies for complete resolution are highlighted by these two cases. Early referral to orthopaedics for surgical intervention to irrigate and debride the joint are necessary to optimize clinical outcomes. Further descriptions of *L. adecarboxylata* infection in immunocompromised children, as well as larger case series in the future, will be key to understanding the complete clinical spectrum in a paediatric population, as well as treatment-related outcomes.
